# Seed dispersal syndrome predicts ethanol concentration of fruits in a tropical dry forest

**DOI:** 10.1098/rspb.2023.0804

**Published:** 2023-07-26

**Authors:** Julia G. Casorso, Allegra N. DePasquale, Suheidy Romero Morales, Saúl Cheves Hernandez, Ronald Lopez Navarro, Kimberley J. Hockings, Matthew A. Carrigan, Amanda D. Melin

**Affiliations:** ^1^ Department of Anthropology and Archaeology, University of Calgary, Calgary, Alberta, Canada; ^2^ Área de Conservación Guanacaste, La Cruz, Costa Rica; ^3^ College of Life and Environmental Sciences, University of Exeter, Exeter, UK; ^4^ Science Department, College of Central Florida, Ocala, FL, USA

**Keywords:** dietary ecology, ecological adaptation, frugivory, phylogenetic signal, sensory ecology, alcohol

## Abstract

Studying fruit traits and their interactions with seed dispersers can improve how we interpret patterns of biodiversity, ecosystem function and evolution. Mounting evidence suggests that fruit ethanol is common and variable, and may exert selective pressures on seed dispersers. To test this, we comprehensively assess fruit ethanol content in a wild ecosystem and explore sources of variation. We hypothesize that both phylogeny and seed dispersal syndrome explain variation in ethanol levels, and we predict that fruits with mammalian dispersal traits will contain higher levels of ethanol than those with bird dispersal traits. We measured ripe fruit ethanol content in species with mammal- (*n* = 16), bird- (*n* = 14) or mixed-dispersal (*n* = 7) syndromes in a Costa Rican tropical dry forest. Seventy-eight per cent of fruit species yielded measurable ethanol concentrations. We detected a phylogenetic signal in maximum ethanol levels (Pagel's *λ* = 0.82). Controlling for phylogeny, we observed greater ethanol concentrations in mammal-dispersed fruits, indicating that dispersal syndrome helps explain variation in ethanol content, and that mammals may be more exposed to ethanol in their diets than birds. Our findings further our understanding of wild fruit ethanol and its potential role as a selective pressure on frugivore sensory systems and metabolism.

## 1. Introduction

Studying the distribution of traits across plants, and the animals that interact with them, allows us to interpret patterns of biodiversity, understand how ecosystems function and evolve, and better implement conservation strategies [1–3]. Angiosperm fruit traits, such as colour, size, odour and hardness, are often associated non-randomly, and correlated traits are hypothesized to have evolved to maximize their attractiveness to different dispersers that possess different sensory and food processing capacities [4–7]. Although the (co)evolution of dispersal syndromes is probably diffuse and may be complicated for some taxa [8–10], support from the literature on non-random trait–frugivore associations suggests it can be a useful concept for exploring the evolution of fruit traits [7,11,12].

Two prominent groups of vertebrate seed dispersers—birds and mammals—differ in their typical activity patterns and the sensory systems they rely on to find and consume fruit [4,13,14]. Avian frugivores tend to be highly visually oriented, with tetrachromatic colour vision based on four cone types, and excellent visual acuity [11,15,16]. They also commonly swallow fruits whole [17,18]. Fruits with ‘bird dispersal syndromes' are correspondingly characterized by small size, absence of a protective husk, and high visual contrast [19–21]. Alternatively, frugivorous mammals, including primates, bats, rodents and ungulates, are typically dichromatic (two-cone vision, red–green colourblind), although monochromacy (single-cone vision, total colourblindness) is found in some nocturnal species, and trichromacy (three-cone vision, human ‘normal’ colour vision) characterizes some primates [22–24]. Additionally, mammalian frugivores often have sensitive hands and mouths [25–28], complex dentition [29], and large olfactory bulbs and olfactory receptor (OR) gene repertoires [30,31]. Reflecting these anatomical features, mammalian frugivores rely less on vision and more on olfaction and manual/buccal haptic sensation and processing during fruit foraging [32–35]. Indeed, fruits with ‘mammal dispersal syndromes' are typically duller and less visually conspicuous, but more odiferous [14,36–39] and can be covered by a thick husk that requires manual or buccal processing to remove.

There is a growing realization that ethanol is pervasive in angiosperm fruits and may exert selective pressures on the sensory and digestive systems of vertebrate frugivores, although this is seldom considered in studies of dispersal syndromes [40–42]. Ethanol occurs naturally in wild fruits and nectars as a product of the fermentation of fruit sugars by naturally occurring yeasts [43]. Accordingly, frugivores may be chronically exposed to low levels of ethanol through their diet [44]. For example, the largest study to date on ethanol consumption by frugivores has revealed that late-stage fermented fruits (those that are likely to contain substantial amounts of ethanol) are a non-negligible component of many non-human-primate diets worldwide [45], and exposure to ethanolic fruits has been hypothesized to shape the evolution of metabolic enzymes across mammals [41,42,46].

We currently know little about the relative concentrations of ethanol in wild fruits, as well as the extent and sources of variation in ethanol concentration. Investigations of ethanol content in frugivore diets have rarely been pursued and are limited to relatively small sample sizes, few regions or anecdotal evidence (see [45,47–52]). Available studies demonstrate that ethanol generally ranges from trace amounts to 2% (vol/vol) [47–52], but can be as high as 8.1% in some fruits [49] and 3.8% in some nectars [53].

The impacts of dietary ethanol may range from beneficial to toxic for frugivores, and different guilds of frugivores may possess adaptations for detecting and processing ethanol. Ethanol may benefit frugivores as it can be a reliable indicator of sugar content, the primary nutritive reward in ripening fruits [50,54,55]. Furthermore, ethanol itself may also be rewarding for frugivores, given that its caloric value is almost twice that of carbohydrates [56]. However, it can also be detrimental as its consumption can impair normal functioning in wild animals [57,58], with deleterious health impacts of chronic ethanol consumption reported, including toxicosis [59,60]. Given its low molecular weight and volatile nature, ethanol may provide an important olfactory cue of sugar content in ripe fruit to foragers [61,62], especially given that some frugivorous mammals are highly sensitive to ethanol odours [62–64]. Selection may favour traits that enable the efficient detection and metabolism of ethanol in frugivores exposed to higher levels of ethanol. Given the olfactory reliance of mammals, the sensitivity of at least some species to ethanol odours, and evidence of genetic adaptations favouring efficient ethanol metabolism in some frugivorous mammals (see [41,42]), fruits primarily dispersed by mammals may exhibit greater ethanol concentration relative to those primarily dispersed by birds [65].

Along with selective pressures from seed dispersers, other factors may shape variation in ethanol concentration across plant taxa. Phylogenetic association, in particular, may be an important factor influencing patterns of ethanol concentration. Angiosperms and ethanol-fermenting yeasts have interacted for millions of years [7,66,67], potentially shaping patterns of yeast interactions and ethanol concentration through phylogenetic relationships. Accordingly, closely related fruit species may exhibit more similar levels of ethanol, resulting in a strong phylogenetic signal. A strong phylogenetic signal in ethanol concentration could reflect similar selective pressures exerted on angiosperms by yeasts, similar yeast communities or similar abiotic conditions, arising from shared ancestry [65,68,69]. The influence of phylogenetic association is well established in ecologically relevant plant traits such as seed size and phenology, in which closely related species tend to have more similar seed sizes and phenological patterns [70,71].

To cultivate a better understanding of the ecological and evolutionary importance of ethanol in the diets of frugivorous vertebrates, and to understand the extent and sources of its variation across plant taxa, we measured ethanol in a large sample of wild fruits consumed by birds and mammals in a Costa Rican tropical dry forest and examined correlates of ethanol concentration. We first build a phylogeny of the plant species we sampled and test for a phylogenetic signal in fruit ethanol concentration. We predict to find an association between phylogenetic relatedness and ethanol concentration. Next, we test the hypothesis that frugivores have influenced the evolution of fruit ethanol production. To do so, we classify fruit species by seed dispersal syndromes and predict that fruits with mammal dispersal traits exhibit significantly higher concentrations of ethanol than fruits with bird dispersal traits.

## 2. Material and methods

### (a) Study site

From December 2019 to December 2020 and from December 2021 to May 2022, we measured ethanol concentration in wild fruits in Sector Santa Rosa (SSR), Área de Conservación Guanacaste, in northwestern Costa Rica (10°45′ to 11°00′ N and 85°30′ to 85°45′ W). SSR is a tropical dry forest with strong climatic seasonality [72]. Annual rainfall ranges between 800 and 2600 mm and maximum temperatures range between 21.6°C and 34.4°C. Most rainfall occurs during the cooler wet season (mid-May to December), while little to no precipitation occurs in the hotter dry season (December to mid-May) [73–76]. The dry forest comprises a diverse and well-studied community of approximately 200 species of angiosperm plants exhibiting various morphologies and dispersal syndromes [77–79]. SSR also hosts a diverse and ecologically intact community of frugivores, including 13 families of frugivorous birds, 10 species of phyllostomid bats, three species of primates, and a variety of terrestrial frugivores including tapirs, agouti, peccaries and deer [80–82].

### (b) Ethanol data collection

We opportunistically collected 70 different fruit species (ranging from six to 24 fruit species per month) over the course of our study. We selected fruits of different ripeness stages (unripe, midripe, ripe, and overripe) for each species whenever possible. Ripeness stages were subjectively assessed using a combination of the palpable softening of fruits, intensity of odour, colour change, fruit size, and evidence of rotting; the subset of traits used for ripeness estimation varied by species. Ripeness estimations were conducted by researchers with extensive experience with these plant species and expertise in tropical dry forest ecology. For instance, to determine the ripeness of fruits covered in a hard husk (e.g. *Psidium guajava*), we relied on colour, odour, and evidence of spoiling. For dehiscent fruits (e.g. *Tabernaemontana odontadeniiflora*), we used the degree to which the husk had opened to expose the arillate seeds. For fruits that did not undergo colour changes upon ripening (e.g. *Ficus morazaniana*), we used odour, size, and softness to assess ripeness. Fruits that showed evidence of losing integrity, shape, or rotting were considered ‘overripe’. We collected fruits directly from the trees by picking fruits off branches or using pruning poles. We also collected fruits from the ground to increase our sample size and to access overripe fruits. Unripe and ripe fruits were only collected from the ground if they were estimated to have fallen in the last 24 h (i.e. we directly observed them fall or be dropped by an animal, or they showed no signs of desiccation or mould, were mostly atop leaf litter, maintained structural integrity, and were not overrun by insects). Overripe fruits were exclusively collected from the ground. The majority of fruits we collected were intact, but we did not require fruits to be perfect (e.g. small scratches and punctures were acceptable). Collected fruits were brought back to the laboratory as soon as possible and their ethanol concentration was measured the same day. We did not alter fruits post-collection prior to measuring ethanol concentration.

To measure fruit ethanol concentration, we customized an ethanol analysis system based around a MARK V breathalyser (Alcovisor, Kwun Tong, Hong Kong, China), a device used to measure blood alcohol level in humans (electronic supplementary material, figure S1). We validated our system by measuring solutions of known ethanol concentration and confirmed measurement accuracy and repeatability. To measure wild fruits, we weighed and placed each sample in a sealed plastic bag filled with at least 120 ml of ambient air (but not more than 100 times the mass of the fruit). If an individual fruit weighed less than 1.2 g, multiple fruits of similar size and ripeness were combined into one bag. All samples were kept in the same location away from direct sunlight for at least one hour to provide sufficient time for ethanol vapours to equilibrate in the headspace; we provide data from a time series experiment in support of this method. These data indicate that ethanol within fruit equilibrates with surrounding headspace in the bag within an hour and remains stable for at least 12 h (electronic supplemental material). Next, we collected 60 ml of air from the headspace using a 16-gauge needle and syringe, and pushed the air in a steady stream through a calibrated MARK V breathalyser. To do this, we fitted the breathalyser with an Alcovisor MARK V mouthpiece and placed a set of overlapping tubes on one end of the mouthpiece. These tubes comprised a 6 in piece of Tygon S3 laboratory tubing (Saint Gobain, Courbevoie, France; inner diameter (ID): 5/32 in, outer diameter (OD): 7/32 in), a 1 in piece of silicone tubing (ID: 3/16 in, OD: 5/16 in), and a 1.5 in piece of silicone tubing (ID: 1/4 in, OD: 3/8 in). The first tube was inserted into the second tube, so the ends were nearly flush. About 0.5 in of the third tube was placed over the breathalyser mouthpiece, and then the first two tubes were inserted into this third tube until their flush ends were near the mouthpiece. With this set-up, the needle on the syringe could be replaced with the tubing and the air from a sample pushed through the breathalyser. The sensor of a MARK V breathalyser is formed by platinum electrodes that react with ethanol vapours to create an electric current proportional to the amount of ethanol in the air [83]. The breathalyser measures air (breath) alcohol concentration (BrAC) and, using the proportional relationship between alcohol levels in the breath and blood in humans, converts this to a measure of blood alcohol content (BAC) [83]. To translate this value to ethanol concentration relevant to our samples, we simultaneously prepared and incubated a series of ethanol standards (5 ml each of water and 0.1%, 0.5%, 1% and 2% alcohol by volume in water, hereafter '%ABV') for each measurement session of fruit samples and measured them under identical conditions to create a standard curve. We used the standard curve to impute %ABV (all ethanol values presented hereafter are in %ABV) from the BAC reading. Between readings, we flushed the breathalyser with fresh air three to five times until it consistently read 0.0. Given that ethanol readings fluctuate with temperature, we stored and measured the standards and fruit samples under the same conditions and measured them sequentially as close together in time as possible to minimize effects of fluctuations in ambient air temperature and humidity.

### (c) Statistical analyses

#### (i) Ethanol measurements

All breathalyser measurements were converted to %ABV using the standard curve generated for that measurement period. For some samples we obtained a small, non-zero BAC reading from the breathalyser (indicating ethanol presence) but generated a zero or negative %ABV imputation value. Given that we did detect ethanol in these samples, we conservatively assigned a value of 10% of the smallest non-zero %ABV value detected in our entire dataset (1.14 × 10^−4^%), giving these samples a very small but positive %ABV value.

Due to the near ubiquitous occurrence of ‘zero’ %ABV values for unripe fruits and ‘zero’ or low values for midripe fruits, as well as the small number of overripe samples, we only analysed measurements taken for ripe fruits. To include a representative sample of ripe fruits in our analyses, we analysed fruits collected from both the trees and the ground, as well as both intact and imperfect fruits (e.g. small scratches or punctures). Of the 70 species we sampled, we only analyse species for which we had sampled at least three ripe fruits in the field (hereafter referred to as adequately sampled species; *n* = 37 species). Our entire dataset, including unripe, midripe, ripe and overripe fruit measurements, is available in our Dryad repository (see Data Accessibility). To determine if three fruits per species provided a representative assessment, we compared the distribution of raw ethanol values for the first three, four, and five ripe fruits sampled for species with at least five ripe fruits sampled (electronic supplementary material, figure S2). For all species, any differences among these raw values were small (electronic supplemental material). We use a threshold of three ripe fruits so that we can include as many species as possible while still having repetition for each species to increase confidence in the values generated.

To aid in visualizing patterns in ethanol concentration across species, we defined categorial ethanol levels by plotting median, mean and maximum ethanol values and visually discerning natural clusters of measurements. Median and mean ethanol values were low due to the large number of ‘zero’ %ABV values in the dataset and did not show distinct clustering. However, using maximum ethanol values, fruit species could be placed into roughly equal-sized groups in the following categories: no (0%), low (less than 0.1%), medium (≥ 0.1% and less than 1%) and high (≥ 1%) ethanol concentration (see electronic supplementary material, figure S3).

#### (ii) Phylogenetic signal

**Figure 1.**
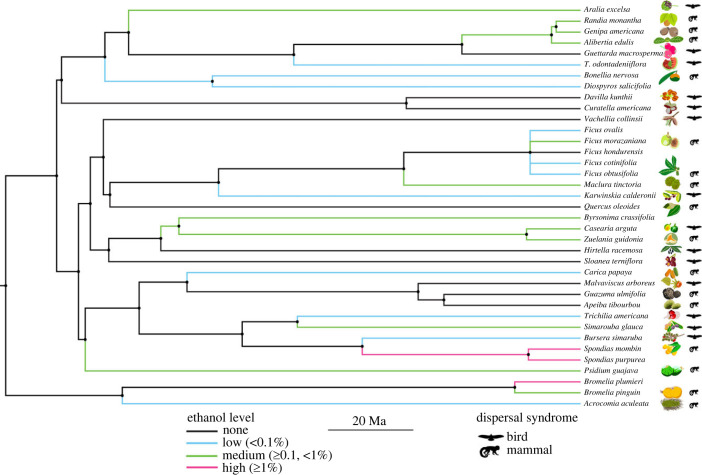
Phylogenetic tree of analysed fruit species sampled in Sector Santa Rosa, Costa Rica (*n* = 37 species). The phylogeny was created using the V.PhyloMaker package and the associated GBOTB mega-tree [86] in R statistical software. Branch colours classified by the maximum ethanol concentration of ripe fruit of each species, as follows. Black: no ethanol concentration ever detected. Blue: low (less than 0.1%ABV detected). Green: medium (≥ 0.1%ABV and <1%ABV detected). Pink: high (≥ 1%ABV detected). When fruits could be assigned to a primary bird/mammal dispersal syndrome, we include the corresponding silhouette and illustration of the fruit. Species without a disperser icon were classified as having a ‘mixed’ dispersal syndrome. *T. odontadeniiflora* is shorthand for *Tabernaemontana odontadeniiflora*. Fruit illustrations by Alyssa Bohart; mammal icon by T. Michael Keesey; bird icon by Andy Wilson.

We used Pagel's *λ* to measure phylogenetic signal in ethanol concentration in wild fruits. This is a widely used metric in evolutionary ecology that has been demonstrated to perform well as a measure of phylogenetic signal [84,85]. We first created a phylogenetic tree of all adequately sampled fruit species collected in this study (*n* = 37 species; figure 1) using the ‘phylo.maker’ function and the mega-tree 'GBOTB.extended’ in the V.PhyloMaker package [86] in R 4.1.1 [87]. The GBOTB mega-tree is a phylogeny derived from two mega-trees [88,89] that includes 79 881 vascular plant taxa. Simulations suggest that polytomies have only negligible impacts on estimates of *λ* [85]. We therefore did not attempt to force pairwise relationships among the few polytomies that arose due to inadequate resolution among sister groups in our phylogenetic tree. Pagel's *λ* relies on a Brownian motion model of evolution, meaning the trait value is assumed to change randomly over time and the expected covariance in trait values between species is directly proportional to their shared branch lengths [84,85]. This model incorporates assumptions of both genetic drift, under which traits evolve randomly, and natural selection, under which selection pressures are continuously changing over time in a constantly fluctuating environment [84]. Using the ‘pgls’ function in the caper package [90] in R 4.1.1 [87] and the phylogenetic tree in figure 1, we separately estimated the value of *λ* for mean and for maximum ethanol concentration across study species. We used likelihood ratio tests to determine if intermediate values of *λ* were significantly different from zero (no phylogenetic signal) or one (complete phylogenetic signal), which helps discriminate between low, intermediate or strong phylogenetic signal [91].

#### (iii) Dispersal syndrome

We classified the dispersal syndrome for each of our sampled fruit species as bird, mammal or mixed dispersal syndrome based on colour, size, structure (i.e. pulp/aril type) and protection morphology following established methods of predicting dispersal syndrome based on fruit traits. We classified fruits as predominantly bird dispersed if they exhibited two or more of the following criteria: brightly coloured or high contrast fruits, including red, pink, bright orange, black, blue/purple, white or mixed-colour fruits; small size (less than 5 g) when ripe; fruits that are dehiscent/unprotected. Conversely, we classified fruits as predominantly mammal dispersed if they exhibited two or more of the following: dull-coloured or low-contrast fruits, including green, yellow, brown or pale orange fruits; large size (≥ 5 g) when ripe; fruits that are protected by a distinct, hard or thick layer that provides a barrier to feeding (e.g. hard husk, spines, thick skin; [5,7,11,14,92–95]). Soft succulent tissue (i.e. fleshy) and the absence of protection (i.e. fruits with a thin, flexible skin) were considered to be mixed-dispersal traits, as both birds and mammals consume such fruits. If a species was consistent with only one or fewer traits specific to bird or mammal dispersal, we considered it to be a mixed-dispersal fruit, and we did not include these species in our analyses. Of our 37 adequately sampled species, we assigned 30 to a specific seed dispersal syndrome: specifically, 16 fruits were considered mammal-dispersed and 14 bird-dispersed (electronic supplemental material, table S1). We were able to verify the majority of our trait-based seed dispersal syndrome categorizations (26/30) by cross-referencing available published work on seed dispersal by birds and mammals in the tropical dry forest (e.g. [77,79,96,97]). Dispersal syndrome categorizations were carried out by authors who were blind to the ethanol data collected. To test whether dispersal syndrome significantly predicts ethanol concentration in wild fruit species, we used a phylogenetic generalized least squares (PGLS) test. This method enables the analysis of trait correlations across species while controlling for potential phylogenetic non-independence [91]. Dispersal syndrome was coded as a binary character (i.e. bird-dispersed (0) or mammal-dispersed (1)) to allow for this trait to be analysed in a PGLS framework. Using the ‘pgls’ function in the caper package [90] in R 4.1.1 [87] and the phylogenetic tree in figure 1 (using only the 30 species that we assigned as either mammal- or bird-dispersed), we analysed the influence of dispersal syndrome on both mean and maximum ethanol concentration across plant species.

## 3. Results

### (a) Phylogenetic signal

We did not detect a statistically significant phylogenetic signal in mean ethanol concentration across our full sample of 37 ripe fruit species. The estimate of Pagel's *λ* for this trait was 0. Conversely, we found a significant phylogenetic signal in maximum ethanol concentration of ripe fruits (figure 1). The estimate of Pagel's *λ* for this trait was 0.82, and likelihood ratio tests indicate that this value significantly differed from a *λ* of 0 (*p* < 0.05) and did not significantly differ from a *λ* of 1 (*p* > 0.05) (i.e. evidence of a significant phylogenetic signal).

### (b) Dispersal syndrome

**Figure 2.**
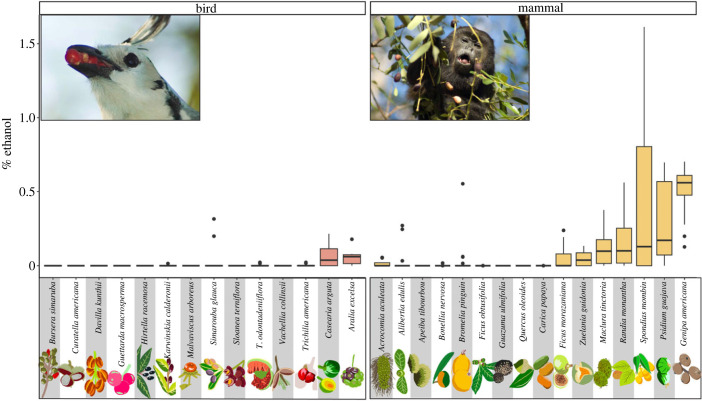
Range of percentage ethanol concentration (% alcohol by volume) of ripe fruits of species sampled at Sector Santa Rosa, Costa Rica (*n* = 37), plotted by seed dispersal syndrome (bird versus mammal). Plant species are arranged in ascending order on the *y*-axis by ethanol concentration. Each boxplot includes the first quartile at the lower hinge, the third quartile at the upper hinge, and the median value (marked by a horizontal bar), with individual data points shown. *T. odontadeniiflora* is shorthand for *Tabernaemontana odontadeniiflora*. Fruit illustrations by Alyssa Bohart. Photo credit: Fernando Campos (magpie jay, *Calocitta formosa,* and howler monkey, *Alouatta paliatta)*.

We tested for an impact of dispersal syndrome in the 30 fruit species for which either bird or mammal syndrome could be clearly assigned. Dispersal syndrome was a significant predictor of both mean ethanol concentration (PGLS, *p* < 0.05; table 1) and maximum ethanol concentration (PGLS, *p* < 0.05; table 1) in ripe fruits. In both cases, we detected significantly higher ethanol concentrations in mammal-dispersed fruits than bird-dispersed fruits (figure 2).

**Table 1.** Summary of phylogenetic least squares regression (PGLS) models testing the effect of dispersal syndrome on mean and maximum ethanol concentration.

	parameter	estimate	std. error	*t*-value	*p*-value
mean ethanol	intercept	0.00012	0.000319	0.3753	0.71028
	disperser	0.00091	0.000437	2.0837	0.04644^a^
maximum ethanol	intercept	0.000551	0.000865	0.6368	0.5294
	disperser	0.002712	0.001184	2.291	0.0297^a^

^a^Denotes statistical significance at *p* < 0.05.

## 4. Discussion

Our aim was to better understand the evolutionary ecology of fruit ethanol concentration by examining its distribution across the angiosperm phylogeny and the extent to which ethanol concentration is predicted by seed dispersal syndromes. We find that mammal-dispersed fruits exhibit significantly higher mean and maximum ethanol levels than bird-dispersed fruits when analysed in a phylogenetic framework. Our findings suggest that frugivorous mammals at our study site are chronically exposed to low levels of ethanol through their diet. We discuss these results in detail below.

### (a) Phylogenetic signal in ethanol concentration of angiosperm fruits

We first examined whether shared evolutionary history shapes fruit ethanol concentration. We found no evidence that the mean ethanol concentration was influenced by the evolutionary history of the plants. While this could indicate either a highly labile or highly stable trait on an evolutionary time scale [91], it is also possible that the nature of the mean ethanol dataset held too little variation to detect a phylogenetic signal. Mean ethanol measurements for ripe fruits were often 0% or very low (e.g. <0.01%), even in species where ethanol concentrations at times exceeded 1%. This led to low variation in the mean values that typically hovered at or near zero. Conversely, maximum ethanol concentration exhibited more variation and we found evidence that this variable is influenced by phylogenetic relatedness, in support of our prediction. While the exact thresholds for ‘weak’ and ‘strong’ phylogenetic signal vary across studies, the high Pagel's *λ* value (0.82) for maximum ethanol is consistent with a strong phylogenetic signal and a trait that evolved under a Brownian motion model of evolution (i.e. changes randomly over time [84,91]). This is similar to other fruit traits, such as phenology and seed size, which also exhibit strong phylogenetic signal [71,98]. This result has interesting implications. It is possible that the fruit properties (e.g. sugar profiles and flesh type) are similar and responsible for the observed phylogenetic similarity. In addition, ethanol concentration is the result of complex eco-evolutionary dynamics between fruits, yeasts, and other microbes. Notably, the metabolic pathway yeasts use to ferment sugars and produce ethanol emerged in the early Cretaceous at approximately the same time that angiosperms shifted from producing small, wind-dispersed fruits to large, fleshy, vertebrate-dispersed fruits with fermentable sugars [66,67]. Future research on the interaction between diverse strains of yeast and the properties of fruits, including relative amounts of different sugars, may further illuminate the factors shaping the evolution of ethanol concentration across fruiting taxa [67,99,100].

### (b) Dispersal syndrome and ethanol concentration

Our second goal was to classify fruit species by seed dispersal syndrome and test the prediction that seed dispersal syndrome explains variation in fruit ethanol concentration. We found that, controlling for phylogenetic relatedness, fruits with mammal dispersal syndromes contain significantly higher mean and maximum ethanol values than bird-dispersed fruits. This is consistent with the hypothesis that seed dispersers shape the plant traits that are involved in ethanol production. It is possible that the larger size (and small surface area to volume ratio) of mammal-dispersed fruits may contribute to the higher ethanol content via allometric effects [44]. It is additionally possible that because our ethanol estimates are derived from headspace sampling of whole fruit, our method further underestimates ethanol values for these larger fruits, which would strengthen the effects we report here. Follow-up study with different methodological approaches, such as those employed by Campbell *et al*. [52], may clarify this. Overall, our results indicate that mammals may be more exposed to ethanol during fruit feeding. Intriguingly, given recent foraging tests and observations, emerging evidence suggests that ethanol may serve as a useful foraging cue for mammals [62,64,101]. Ethanol odour plumes may aid foraging mammals as a short-distance cue during fruit assessment and selection, and further may join other volatiles to facilitate locating ripe fruit crops over longer distances via chemotaxis [49,102,103]. However, at least two studies suggest that in large concentrations (greater than 1%) ethanol may act as a feeding deterrent to some species, suggesting a functional range for attractive concentrations that could be explored in future research [51,104].

Ethanol increases as fruits ripen, suggesting that ethanol odours may serve as a reliable indicator of the increased nutritional (typically sugary) reward present in mature fruits to foraging mammals [49–51,62,102]. Fruit aromas are complex mixtures of volatile organic compounds that vary from species to species [105,106]. Our finding that ethanol concentration is rather pervasive across angiosperms suggests exposure to naturally occurring fruit ethanol could therefore apply a strong selective pressure in favour of traits that enhance the ability to detect and metabolize ethanol, as discussed at length in Dudley [44]. This idea is supported by evidence that some frugivorous mammals, including African elephants, squirrel monkeys, pigtail macaques and aye-ayes, are sensitive to ethanol odours [62,64,101]. Further, there is widespread variation in the ability of mammals to metabolize ethanol, with species whose diets consist of greater than 50% fruit/nectar exhibiting intensified selective pressures acting on underlying alcohol dehydrogenase (*ADH7*) genes [42]. Indeed, some mammals, including humans, African great apes and aye-ayes, exhibit a mutation in their *ADH7* gene that yields a 40-fold improvement in enzymatic efficiency for ethanol metabolism, and these animals may have significant exposure to dietary ethanol [41,42,107].

While ethanol has traditionally been viewed as a feeding deterrent to frugivores, and yeasts have traditionally been viewed as competitors with frugivores for fruit sugars (i.e. [108]), a number of more recent studies reject this hypothesis and suggest that behavioural responses to ethanol may be nonlinear [52]. Ethanol may be attractive at low concentrations and aversive at higher concentrations [109]. Further, the relationship between fruit, yeasts and vertebrate frugivores may represent a tripartite mutualism [65]. Colonization by yeasts is posited to make fruits more attractive to vertebrate frugivores, who may use ethanol and ethanol-associated odours to forage efficiently [50,61]. These frugivores then provide dispersal services to both the fruit seeds and the yeast spores [69]. Our study furthers this idea by providing some of the first evidence that fruits that are likely to be predominantly dispersed by mammals contain more ethanol than bird-dispersed fruits. This may exert selective pressures on the fruits, and also on the sensory and digestive anatomy of mammals that is adaptive for detecting, locating and metabolizing ethanol. This idea invites rigorous testing in future comparative studies.

It is important to also highlight limitations of our study. The breathalyser method we employ here has numerous advantages, including its user-friendly, field-portable design; however, there are limitations imposed by utilizing a headspace-based approach, rather than directly measuring pulp ethanol concentrations. Our method probably underestimates pulp ethanol concentration, especially for larger, mammal-dispersed fruit, which may be greater than the concentrations we present here. If so, this would further strengthen our conclusions. We also did not directly measure the ethanol concentrations of the different fruit parts consumed by the frugivores. Such an approach (e.g. [52]) may yield additional insights and allow more comprehensive assessment of our headspace vapour-based method. Finally, our study is limited by only having sufficient sample size to statistically analyse ripe fruit. Future research could usefully examine a larger sample of fruits across ripeness stages and longitudinally track ethanol concentrations within fruits, as overripe fruit may be significantly more ethanolic [45].

## 5. Conclusion

We surveyed naturally occurring fruit ethanol across the angiosperm phylogeny in a Costa Rican tropical dry forest and we provide clear evidence that seed dispersal syndrome predicts mean and maximum ethanol concentration when controlling for phylogenetic relatedness. In doing so, we help to fill a persistent knowledge gap on the concentrations of naturally occurring fruit ethanol and we suggest that mammalian frugivores at our site are chronically exposed to low levels of dietary ethanol. This research is among the first to survey ethanol concentrations across a relatively large sample of fruit species and we find a clear phylogenetic signal in maximum ethanol concentration. Nevertheless, we still know little about mechanisms shaping variation in ethanol concentration or about the distribution of ethanol across angiosperms globally and the potential for biogeographic differences in ethanol concentration. Future work could benefit by explicitly incorporating measures of sugar concentration and yeast strains into data collection, by exploring co-variation between fruit traits and ethanol levels, and by assessing impacts of climatic seasonality and habitat variation across a broader geographical area. Such a research programme could shed further light on the behavioural, physiological and anatomical adaptations frugivores use to find and consume ethanol. These investigations are fundamental to our understanding of the broader historical and contemporary eco-evolutionary dynamics occurring between fruit, yeasts and frugivores.

## Data Availability

Data and code to reproduce our analyses, as well as our full dataset, are available on Dryad (https://doi.org/10.5061/dryad.bzkh189fm) [110]. Additional information is provided in electronic supplementary material [111].
